# Assessing patterns of extinction risk amongst mammal species in Nigeria: A comparative analysis of human impact

**DOI:** 10.3897/BDJ.14.e191439

**Published:** 2026-08-21

**Authors:** Bernard Asanbe

**Affiliations:** 1 University of Gothenburg, Göteborg, Sweden University of Gothenburg Göteborg Sweden

**Keywords:** brain mass, extinction risk, human impact, Nigerian mammals

## Abstract

This study aimed to evaluate how biological traits influence extinction risk amongst mammal species in Nigeria and how these traits interact with specific anthropogenic threats, such as agriculture, urbanisation and climate change. Focusing on mammal species in Nigeria, we used phylogenetic logistic regression to test the influence of five biological traits: body mass, brain mass, generation time, current geographic range and historical range contraction, on extinction risk across nine IUCN threat categories. Standardised models were used to compare trait sensitivity across threats. Brain mass emerged as the most consistent and influential predictor of extinction risk across threat categories, including agriculture, biological resource use, urban development and, notably, climate change, where it was the strongest predictor of all models. Species with larger brains, often primates and carnivores, were highly vulnerable. Geographic range size was a strong negative predictor of risk across most models, with range-restricted species more susceptible to habitat loss and fragmentation. Generation time was positively associated with risk under direct human pressures, but inversely linked under climate threats. Body mass showed weak and inconsistent effects overall, though it was significantly and negatively associated with extinction risk under agriculture and aquaculture. The number of species affected was highest under direct human pressures, compared to indirect anthropogenic threats, such as climate change and pollution. Extinction risk in Nigerian mammals is shaped by intrinsic traits that interact predictably with human pressures. Species with large brains, small ranges and slow reproduction are at greatest risk. Trait-based models can improve conservation planning by identifying vulnerable species before population declines become critical, especially in regions facing intensive land-use change.

## Introduction 

The rate at which global biodiversity is declining presents significant environmental, economic and social challenges, which continue to result in extinction risk levels rising at unprecedented scale due to human activities in this present age (Estrada et al. 2017). This decline is typically observed in regions that are known for their high levels of species richness and vulnerability to anthropogenic impacts (Pereira et al. 2012). According to the current International Union for Conservation of Nature’s Red List database, over 46,300 species are threatened with extinction globally (IUCN 2024). This includes examples such as the Javan rhinoceros, African elephant, western gorilla and chimpanzee. Not only are species with limited geographical range, large body mass, large brain mass and low reproductive rates confronted with increased extinction risk, they are also faced with threats due to human activities, habitat degradation, climate change and invasive species (Maxwell et al. 2016).

Mammals which play important roles within ecosystems, such as predators, prey and seed dispersers, are increasingly threatened with approximately 26% of its species at risk of extinction (IUCN 2024). In Nigeria, mammal species are facing significant extinction risk due to human activities, such as agriculture, urbanisation, poaching and deforestation, which have continuously degraded their habitats (Anwadike 2020). Being part of the Africa's Guinean-forest biodiversity hotspot, the Nigerian lowland forest region is particularly vulnerable to these threats (Ukpoju et al. 2023).

This study offers the possibility to identify specific traits linked to extinction risks amongst mammal species in Nigeria. Species with smaller geographical ranges, such as the chimpanzee, are usually vulnerable due to habitat fragmentation from deforestation and agricultural development (Sesink et al. 2015). Large-bodied mammals such as elephants are at high risk because they require extensive habitats and are frequently in conflict with human activities (Montero-Botey et al. 2024). Larger brained mammals, such as Olive baboons and Leopards (primates and carnivores), are often associated with enhanced cognitive abilities and behavioural complexity, traits that can improve survival in natural environments, but may also increase sensitivity to anthropogenic changes (Abelson 2019, Chichorro et al. 2019).

Despite Nigeria region's richness in biodiversity, studies on extinction risk, especially on mammalian populations are limited, thereby creating a knowledge gap in conservation science and intervention in the region (Imarhiagbe et al. 2019, Ukpoju et al. 2023, Gbadegesin and Gbadamosi 2024). The study addresses this gap by assessing what biological traits increase the vulnerability of Nigerian mammal species to extinction and how do these interact with specific human caused threats?

The study aims to examine extinction risk patterns amongst Nigerian mammals to identify traits that correlate with higher susceptibility to extinction, in order to provide data-driven insights that can provide information for conservation policies, particularly in regions where resources for biodiversity protection are limited and need to be prioritised effectively.

## Material and methods

**Study design and data compilation**

We compiled data from several trusted sources, including the IUCN Red List, phylogenetic trees and trait databases (Faurby et al. 2018, Upham et al. 2019, IUCN 2024). We focused on biological traits that are known to influence extinction risks specifically, body mass, brain mass, generation time (as a proxy for reproductive rate) and geographic range (Chichorro et al. 2019, Healy et al. 2019).

The first dataset we downloaded and compiled was a comprehensive dataset of mammalian species in Nigeria from the IUCN Red List database. The downloaded raw spatial data in the form of a polygon shapefile (SHP) was processed in RStudio software (Lander 2014). The SHP file was processed in RStudio using the *sf* and *tidyverse* packages (Wickham 2014) to extract species attribute data from the spatial polygons and export it to a CSV file for final cleaning in Microsoft Excel, yielding 314 mammalian species in Nigeria observations.

Species names from the IUCN Red List (SCI_NAME column) were matched against mammalian taxonomies from two phylogenetic databases; PHYLACINE 1.2 (Faurby et al. 2018) and a credible set of phylogenetic trees from Upham et al. (2019), using VLOOKUP in Excel. Mismatches and outdated names were verified against the IUCN Red List and relevant taxonomic references and updated to reflect recent revisions (Moura et al. 2024), yielding a harmonised species list of 314 taxa.

To better understand extinction risk, we incorporated additional datasets into the IUCN species data. These include biological traits (Body mass, Brain mass and Reproductive rate), Geographic Range (in km^2^) and the hierarchical structure of the threat types (IUCN 2025).

Body mass data were obtained from the PHYLACINE 1.2 database (Faurby et al. 2018) and brain mass and generation time data from the COMBINE database (Soria et al. 2021). Trait columns were renamed for consistency and merged with the primary dataset using species scientific names as identifiers. Taxonomic mismatches were resolved manually by inferring values from close relatives or genus-level means in a small number of cases (see Limitations). Geographic range sizes were extracted from the IUCN Red List spatial polygon data and calculated in R using the sf package (Pebesma 2018), following the steps described below.

Geographic range areas were calculated from the IUCN polygon shapefiles using the *expanse()* function in the *terra* R package (Hijmans 2024), applied to the original WGS84 (EPSG:4326) coordinate reference system with the transform = TRUE argument to ensure accurate area estimation across the full extent of Nigeria. Three area variables were derived for each species: total geographic area (extant and extinct polygons combined), current area (extant polygons only: 'Extant (resident)', 'Extant & Reintroduced (resident)' and 'Possibly Extant (resident)') and extinct area. Range data could not be computed for 17 species, primarily aquatic or semi-aquatic mammals, due to missing polygon data in the IUCN shapefile; these were retained with a value of zero for consistency.

We additionally integrated into this dataset, the full hierarchical structure of the threat types (IUCN 2025) as listed in the IUCN Threats Classification Scheme (Version 3.3). This dataset was constructed classifying the mammals as threatened or not-threatened, based on their assessed conservation status according to the classification scheme.

**Threat classification data**

Threat exposure data were accessed manually from individual IUCN 2025 Red List species profiles. For each of the 12 IUCN threat classes (Version 3.3), species were assigned a binary value of 1 (threatened by that class) or 0 (not threatened), recorded in Yes/No format during data entry and subsequently coded as 1/0 for modelling. A value of 1 was assigned if the threat appeared on the species profile at any severity or scope; 0 indicated the threat was not listed. For species listed under multiple threats, all applicable classes were coded as 1. No prioritisation of primary threats was performed. All 314 observations were cross-checked against the IUCN Threats Classification Scheme for consistency.

**Data and statistical analysis**

The dplyr and tidyverse packages (Wickham et al. 2023) were utilised for data loading, cleaning and transformation, ensuring only relevant traits were extracted and merged (Wickham 2014). We normalised all these continuous trait variables using the R *scale()* function on only the CurrentArea_TotalArea variable, while we used the R *scale(log10())* function on Body_Mass, Brain_Mass, Current_Area and Generation_time. We use the *log10()* function in R to normalise trait variables by first reducing skewness and stabilising variance through log-transformation and then *scale()* function to standardise them to have a mean of zero and standard deviation of one, ensuring comparability across predictors in regression models. In addition to current range size, we calculated a measure of historical range contraction for each species by dividing its current geographic range (Current_Area_KM2) by its total historical geographic range (Total_Geo_Area_KM2). The CurrentArea_TotalArea ratio, hereafter referred to as historical range contraction, ranges from 0 to 1, with lower values indicating greater cumulative habitat loss relative to the historical range. This variable was scaled using the R *scale()* function (without log-transformation, as it is a bounded ratio; see Warton and Hui (2011)) and included as a predictor in all models.

The link to my openly available dataset that underpins my work is: https://doi.org/10.5281/zenodo.20646160.

We constructed a series of phylogenetic logistic regression models to examine the relationship between biological traits and extinction risk amongst Nigerian mammals (Ives and Garland 2010). We used a set of five key trait variables in the modelling process, all of which were previously log-transformed and scaled. These variables are:

1. Scaled_CurrentArea_TotalArea;
2. Scaled_Log_Brain_Mass_g;
3. Scaled_Log_Current_Area_KM2;
4. Scaled_Log_Generation_time_d;
5. Scaled_Log_Mass_g.

We tested all possible combinations of the five trait predictors across models and applied multicollinearity diagnostics to refine the final models. To do this, we calculated Variance Inflation Factor (VIF) scores in *car*; a R package (Fox and Weisberg 2019), for all trait combinations and excluded those with VIF > 2 to minimise multicollinearity. This step ensured the statistical independence of predictors. Using these five traits, we constructed comprehensive phylogenetic logistic regression models (Ives and Garland 2010) that included combinations of the traits against extinction risk across 12 IUCN threat categories derived from the Threats Classification Scheme (Version 3.3). These threat categories initially included:

1. Residential & commercial development (n = 57 species);
2. Agriculture & aquaculture (n = 106);
3. Energy production & mining (n = 38);
4. Transportation & service corridors (n = 28);
5. Biological resource use (n = 169);
6. Human intrusions & disturbance (n = 33);
7. Natural system modifications (n = 23) — **excluded**;
8. Invasive and other problematic species, genes & diseases (n = 16) — **excluded**;
9. Pollution (n = 28);
10. Geological events (n = 0) — **excluded**;
11. Climate change & severe weather (n = 36);
12. Other options (n = 3) — **excluded**.

Above, “n” refers to the number of mammal species in the dataset that we classified as being threatened in each specific IUCN threat category. We excluded categories 7, 8, 10 and 12 with fewer than 25 observations because models with very small sample sizes (e.g. fewer than five observations per predictor variable) lack sufficient statistical power, increase the risk of overfitting and produce unreliable or unstable estimates in logistic regression analysis.

In total, we retained and ran nine separate phylogenetic logistic regression models. We constructed the nine separate phylogenetic logistic regression models; one Total Threats Model, based on overall IUCN Red List status and eight threat-specific models; using the *phyloglm()* function (method = '*logistic_MPLE*') from the phylolm package, with the pruned phylogenetic tree incorporated via the ape package to account for shared evolutionary history amongst species (Ives and Garland 2010, Ho and Ané 2014, Paradis et al. 2004).

**Visualisation of model outputs**

To visually represent the effect sizes of biological traits across different threat models, we utilised the *ggplot2* package in R (Wickham 2009), a widely used tool for data visualisation in scientific research. Other R packages used alongside *ggplot2* include, *dplyr, readr* and *scales* (Wickham 2009, Wickham et al. 2023). We loaded the visualisation dataset into R and we added significance labels, based on p-values by computing vertical position for the asterisk annotations. To avoid overlapping with the bars, we computed vertical offsets for significance indicators (asterisks), based on each trait’s effect size and standard error. We annotated significance levels using conventional asterisk notation (***p < 0.001, **p < 0.01, *p < 0.05), reflecting standard p-value thresholds.

**Figure 1.**
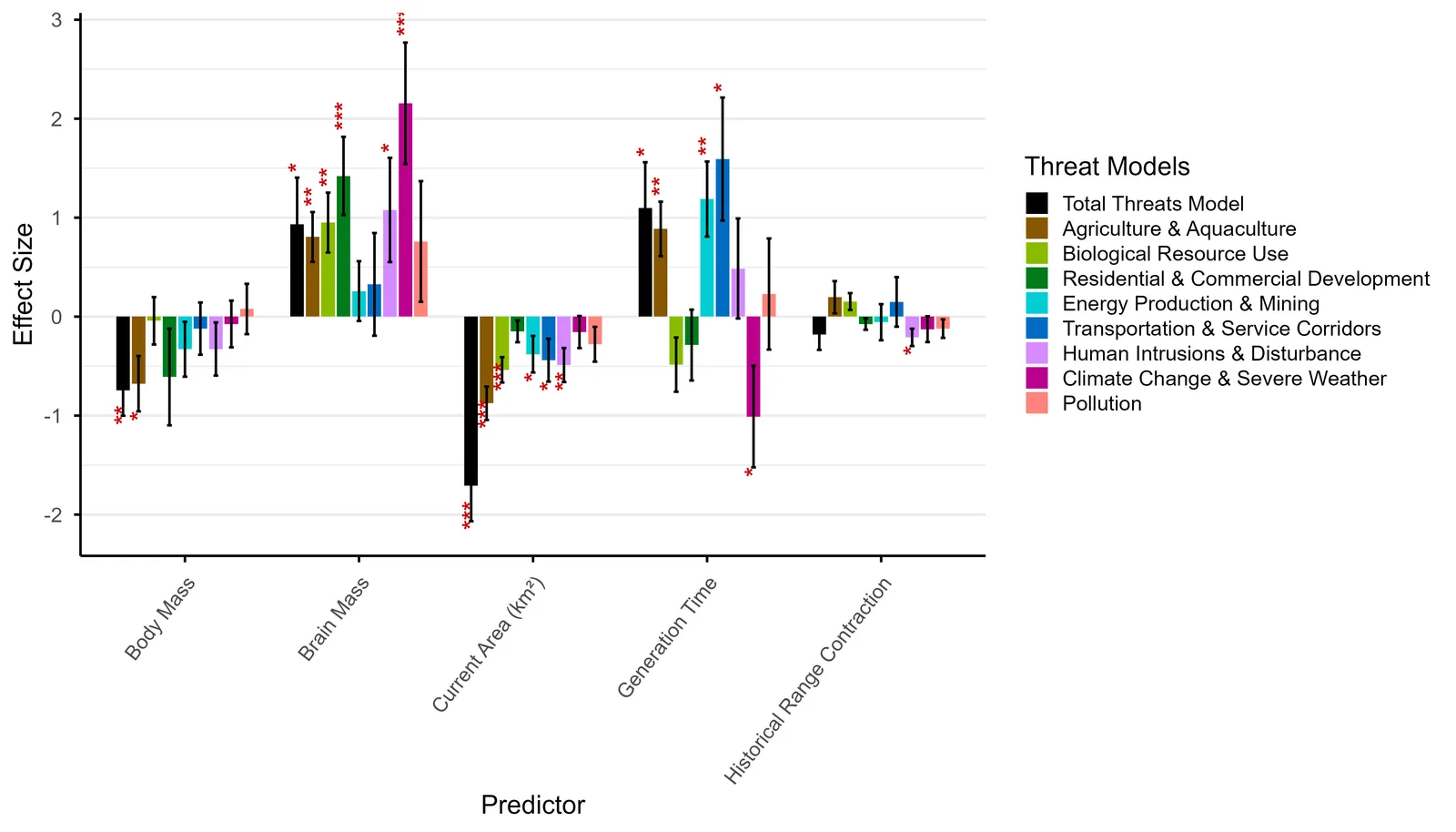
Effect sizes of biological trait predictors across IUCN threat models. Bars represent regression coefficients (± SE) from phylogenetic logistic regression models. Asterisks indicate significance: ***p < 0.001, **p < 0.01, *p < 0.05. Colours represent different threat models; the black bar indicates the Total Threat Model.

**Figure 2.**
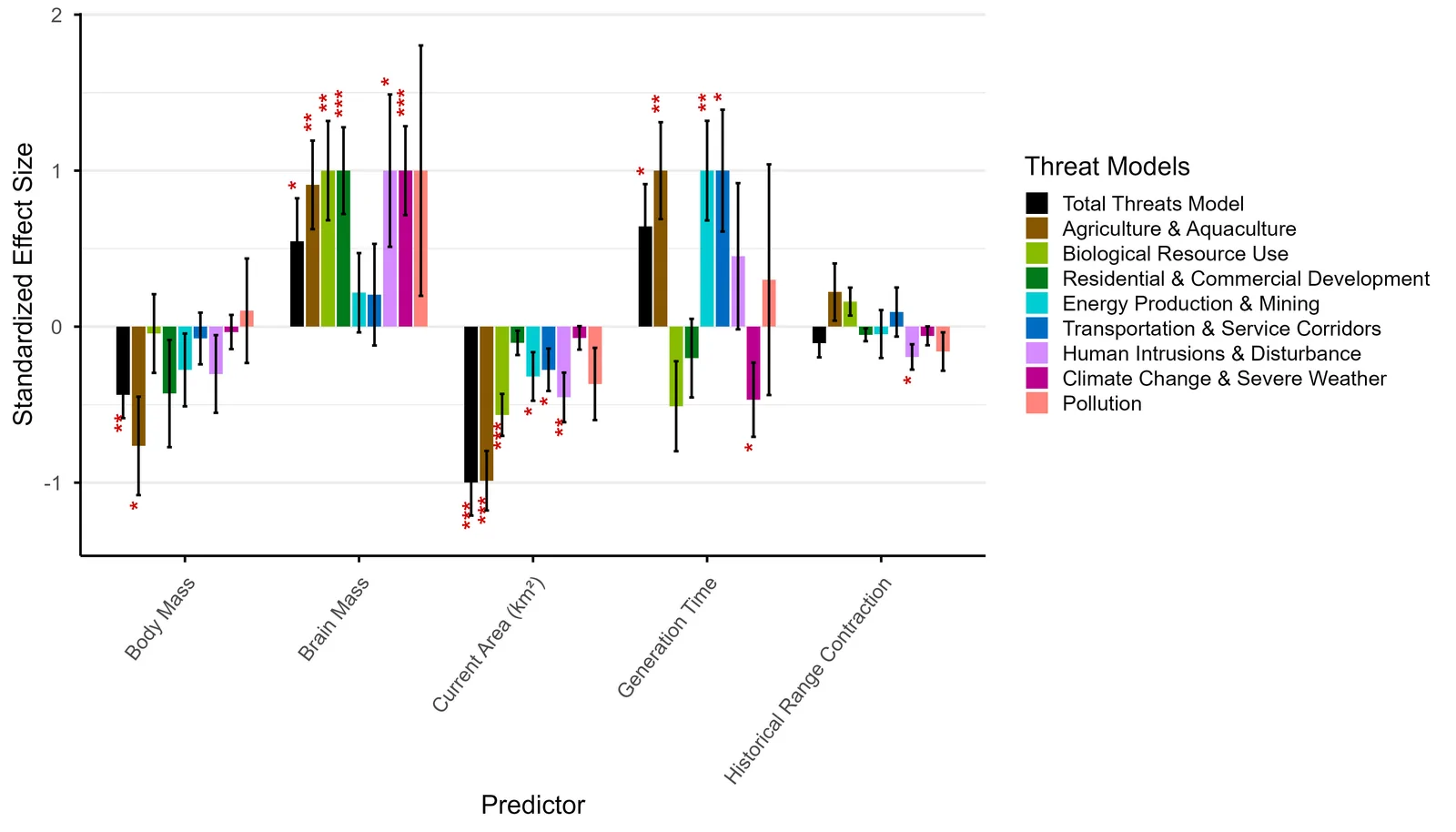
Standardised effect sizes (Std_SE) of trait predictors across threat models. All predictors are standardised (mean = 0, SD = 1) to allow direct comparison of effect sizes across traits. Bars indicate standardised regression coefficients derived from phylogenetic logistic regression. Predictors include current-area by total-area, brain mass, current area, generation time and body mass. Asterisks indicate significance (***p < 0.001, **p < 0.01, *p < 0.05). Colours represent different threat models; the black bar indicates the Total Threat Model.

We ordered threat models for interpretability and applied a high-contrast colour palette, with the Total Threats Model highlighted in black for emphasis. We generated the final plot by creating bar plots with error bars to display the direction, magnitude and precision of trait effects across each phylogenetic logistic regression model. We formatted plot titles, axis labels and legends for clarity and consistency. We applied this computation to generate both the raw effect size plot (Fig. 1) and the standardised effect size plot (Fig. 2).

## Results

**Trait effects across threat models**

Amongst all the models we evaluated, the Total Threat Model (Fig. 1, black bars) provided the broadest insight into extinction risk across Nigeria’s mammalian fauna. This model identified the small current geographic range as the most robust predictor. Larger brain mass also emerged as a strong predictor. Generation time showed a statistically significant positive association with extinction risk in this model. Body mass was significantly and negatively associated with risk. These results are visualised in Fig. 1, which summarises trait effects across all threat models.

Four threat-specific models stood out: Agriculture & Aquaculture, Biological Resource Use (hunting), Residential & Commercial Development and Climate Change & Severe Weather. In the agriculture model, extinction risk was significantly higher amongst species with longer generation times, larger brains and smaller range sizes. Similarly, the best model for hunting, which is the Biological Resource Use model, shows that brain mass and range size were significant, reflecting patterns of targeted extraction in species with higher cognitive or social traits.

In the Residential & Commercial Development model, brain mass was again a highly significant predictor. Although other traits like body mass and range size were also negatively associated with risk, they did not reach statistical significance. Other models, such as Energy Production & Mining, Transportation and Climate Change & Severe Weather, also revealed meaningful trait associations, particularly with generation time and range size. For instance, species with longer generation times were at significantly higher risk from infrastructure development and mining activities, while brain mass was a key vulnerability factor under climate-related threats.

Brain Mass was the most consistent and influential predictor across models (Fig. 2), with the highest standardised effect sizes in Climate Change & Severe Weather, Residential & Commercial Development and Biological Resource Use. Its strong and repeated significance suggests that larger-brained species may be more vulnerable. Current Area (km²) exhibited consistently negative effects and was highly significant in Agriculture & Aquaculture, Biological Resource Use, Human Intrusions and the Total Threats Model. These results highlight that species with limited geographic ranges are particularly susceptible to a variety of threats.

Generation Time showed strong positive associations in Energy Production & Mining, Agriculture & Aquaculture and Transportation & Service Corridors, implying that species with longer life cycles and slower reproduction are more at risk under rapid anthropogenic change. Notably, under Climate Change & Severe Weather, the direction of this relationship reversed, with shorter generation times associated with greater extinction risk, suggesting that fast-reproducing species may be more physiologically sensitive to rapid environmental change, though this effect was weaker than those observed under direct human pressures.

Body mass had generally weak effects, but was a statistically significant negative predictor under Agriculture & Aquaculture, indicating that larger-bodied species face elevated extinction risk under agricultural land-use pressure, consistent with the well-documented vulnerability of large mammals to habitat conversion. Historical range contraction showed limited predictive power overall. It was the only significant association under Human Intrusions & Disturbance, where species that had lost a greater proportion of their historical range were more vulnerable. The predictor was not significantly associated with overall extinction risk in the Total Threats Model, nor with any other threat-specific model, suggesting it has less consistent predictive utility than absolute current range size.

## Discussion

Brain mass emerged as the most consistent and statistically significant predictor in this study across multiple threat categories. This result was particularly evident under threats such as Residential & Commercial Development, Agriculture & Aquaculture, Biological Resource Use and Climate Change & Severe Weather (Fig. 1). In all cases, larger brain mass was positively associated with increased extinction risk. This supports earlier findings that large-brained species, especially primates, tend to be more behaviourally specialised and are extremely affected by human-induced pressures (Gonzalez-Voyer et al. 2016, Chichorro et al. 2019). Larger brains are often associated with traits such as longer developmental periods, delayed reproduction and higher energy demands (Gonzalez-Voyer et al. 2016). These traits, although beneficial in stable environments, may impair resilience in the face of rapid anthropogenic change. Furthermore, such species are often the targets of wildlife trade and hunting (Maxwell et al. 2016), exacerbating their vulnerability.

Species with smaller current geographic ranges were significantly more at risk under Agriculture & Aquaculture, Biological Resource Use, Human Intrusions and in the Total Threats Model. This aligns with well-established evidence that range-restricted species are particularly susceptible to extinction (Slatyer et al. 2013, Sesink et al. 2015). In Nigeria, habitat loss from agriculture and logging has disproportionately impacted forest-dwelling species, many of which already occupy limited ranges in the Guinean forest zone (Luiselli et al. 2019, Maxwell et al. 2016). Our findings reinforce the critical role of spatial extent in mediating species persistence, particularly under land-use pressure (Halley et al. 2014).

A longer generation time predicted higher extinction risk under threats such as Agriculture & Aquaculture, Energy Production & Mining and Transportation & Service Corridors (Fig. 1). This aligns with life-history theory, which suggests that slow-reproducing species recover more slowly from population declines (Gaillard et al. 2000, Robbins and Sawyer 2007). However, under Climate Change & Severe Weather, the relationship was reversed, with shorter generation times associated with greater risk. This suggests that reproductive strategy interacts differently with threat type, possibly due to fast-living species being more sensitive to environmental variability (Chichorro et al. 2022).

Body mass displayed weak or non-significant effects in most models, reaching statistical significance only in Agriculture & Aquaculture. While larger body size is often linked to increased extinction risk due to higher energetic demands and larger home range requirements (Cardillo et al. 2005, Chichorro et al. 2019, Montero-Botey et al. 2024), this relationship appears to be less robust than for brain mass or range extent in the Nigerian context. This may reflect indiscriminate human pressures on both small and large species from hunting and habitat loss (Fa and Brown 2009), the historical depletion of large-bodied fauna (Faith 2014) and the stronger predictive influence of cognitive and spatial traits (Sol et al. 2002, Sesink et al. 2015).

The extent of range contraction, quantified as the ratio of the current to the historical geographic range, showed limited explanatory power across models. It was a significant predictor only under Human Intrusions & Disturbance, where species with greater cumulative range loss were more vulnerable. It was not significantly associated with the Total Threats Model (Fig. 1). Compared to absolute current range size, which was a consistently strong negative predictor across multiple models, historical range contraction appears less informative, possibly because variability in historical range estimation reduces its precision or because current range size more directly captures contemporary habitat availability (Luiselli et al. 2019, Montero-Botey et al. 2024).

**Conservation implications**

Large-brained species, such as primates and carnivores, should be prioritised due to their elevated risk under multiple threats. Many of these species are already targets of hunting and trade (Fa et al. 2002, Luiselli et al. 2019). Species with small geographic ranges require focused habitat protection, especially in unprotected or highly fragmented areas. Long generation species, often large mammals, face increased risk under slow landscape recovery conditions and may benefit from population management and long-term monitoring.

**Limitation**

While this study provides valuable insights into the trait-based determination of extinction risk amongst Nigerian mammals, several limitations need to be mentioned. Foremostly, the IUCN threat data used to classify species exposure to different threats may have limited resolution or attribution accuracy, especially for indirect threats such as pollution and climate change (IUCN 2024). These threats often act gradually or through complex pathways, making it difficult to establish direct cause-effect relationships (Maxwell et al. 2016). As a result, trait-risk associations under these categories may be less precise. Second, although trait data were obtained from robust global databases, a small subset of species required manual correction or estimation due to missing values or taxonomic mismatches. Body mass was directly available for 312 of 314 species (99.4%) from PHYLACINE 1.2. Brain mass and generation time data were drawn directly from the COMBINE database for 295 species (93.9%), with values inferred from close phylogenetic relatives for 16 species (5.1%) and imputed for three species (1.0%). Across all traits, fewer than 7% of values required manual estimation, which may introduce minor uncertainty into model estimates for those species (Penone et al. 2014). The overall quality and consistency of the trait data are considered adequate for the purposes of this analysis. Finally, while the phylogenetic logistic regression framework helps correct for shared evolutionary history, results are inherently shaped by the completeness and accuracy of the phylogenetic tree and underlying species taxonomy (Rabosky 2016).

## Conclusions

This study provides evidence that extinction risk amongst Nigerian mammal species is not random, but biologically patterned. This suggests that certain biological traits, particularly brain mass, geographic range and generation time, consistently make species more vulnerable. Species with larger brains, notably primates and carnivores, were consistently more vulnerable across multiple threat categories, such as agriculture, biological resource use, urban development and, exceptionally, climate change. This vulnerability likely stems from their behavioural specialisation, low reproductive output and increased interaction with human-altered environments (Gonzalez-Voyer et al. 2016, Chichorro et al. 2019). Similarly, we found species with restricted geographic ranges to be significantly at risk due to their limited adaptability and heightened sensitivity to localised disturbances, a pattern well-documented in biodiversity hotspots like Nigeria’s Guinean forest region (Maxwell et al. 2016, Luiselli et al. 2019).

While longer generation time increased risk under direct anthropogenic pressures, its relationship with extinction risk varied under climate-related threats, where short-lived species appeared more susceptible, possibly due to their physiological sensitivity to rapid environmental change (Gonzalez-Voyer et al. 2016). In contrast, body mass, often highlighted in global extinction studies, showed weak and inconsistent effects in this study. This may suggest that in the Nigerian context, both large and small mammals may be equally impacted by these pressures or that other traits, such as cognitive ability and spatial range, offer greater predictive strength. Although climate change showed a particularly strong trait-based effect on brain mass, pollution exhibited little to no significant trait associations, possibly reflecting more diffuse, indirect mechanisms or limitations in threat attribution (Rhind 2009). These findings reinforce the need for localised trait-based analyses, as extinction dynamics can diverge meaningfully from broader global patterns.

Our findings support the integration of trait-based and phylogenetically informed models into conservation planning, which is even more critical in under-represented regions like Nigeria, where data-driven strategies are urgently needed. Prioritising species with high trait-based vulnerability, such as those with large brains, small ranges and slow reproduction, could improve the efficiency of conservation efforts (Kumschick and Richardson 2013). In addition, the study underscores the value of tailoring responses to specific threats, as different drivers filter species along different biological axes. This research enhances our understanding of extinction dynamics in a key region of African biodiversity hotspots and based on intrinsic species biological traits; it provides a scalable framework for guiding conservation interventions.

## References

[B13952797] Abelson E. S. (2019). Big brains reduce extinction risk in Carnivora. Oecologia.

[B13952815] Anwadike B. (2020). Biodiversity conservation in Nigeria: perception, challenges and possible remedies. Current investigations in agriculture and current research.

[B13952824] Cardillo M., Mace G. M., Jones K. E., Bielby J., Bininda-Emonds O. R., Sechrest W., Purvis A. (2005). Multiple causes of high extinction risk in large mammal species. Science.

[B13952837] Chichorro F., Juslén A., Cardoso P. (2019). A review of the relation between species traits and extinction risk. Biological Conservation.

[B13952846] Chichorro F., Correia L., Cardoso P. (2022). Biological traits interact with human threats to drive extinctions: A modelling study. Ecological Informatics.

[B13952855] Estrada A., Garber P., Rylands A., Roos C., Fernandez-Duque E., Fiore A., Nekaris K., Nijman V., Heymann E., Lambert J., Rovero F., Barelli C., Setchell J., Gillespie T., Mittermeier R. (2017). Impending extinction crisis of the world's primates: why primates matter. Science Advances.

[B13952920] Faith J. T. (2014). Late Pleistocene and Holocene mammal extinctions on continental Africa. Earth-Science Reviews.

[B13952886] Fa J. E., Peres C. A., Meeuwig J. (2002). Bushmeat exploitation in tropical forests: an intercontinental comparison. Conservation biology.

[B13952895] Fa J. E., Brown D. (2009). Impacts of hunting on mammals in African tropical moist forests: a review and synthesis. Mammal Review.

[B13952875] Faurby S., Davis M., Pedersen R., Schowanek S., Antonelli A., Svenning J. (2018). PHYLACINE 1.2: The Phylogenetic Atlas of Mammal Macroecology. Ecology.

[B13952912] Fox J., Weisberg S. (2019). An R Companion to Applied Regression.

[B13952929] Gaillard J. M., Festa‐Bianchet M., Yoccoz N. G., Loison A., Toïgo C. (2000). Temporal variation in fitness components and population dynamics of large herbivores. Annual Review of Ecology, Evolution, and Systematics.

[B13952939] Gbadegesin O., Gbadamosi O. (2024). Indigenous ecological knowledge: a transformative approach to biodiversity legislation in Nigeria. Environmental Management.

[B13952957] Gonzalez-Voyer A., Gonzalez-Suarez M., Vilà C., Revilla E. (2016). Larger brain size indirectly increases vulnerability to extinction in mammals. Evolution.

[B13952966] Halley J. M., Monokrousos N., Mazaris A. D., Newmark W. D., Pantis J. D. (2014). Dynamics of extinction debt across five taxonomic groups. Proceedings of the National Academy of Sciences.

[B13952976] Healy K., Ezard T. H.G., Jones O. R., Salguero-Gómez R., Buckley Y. M. (2019). Animal life history is shaped by the pace of life and the distribution of age-specific mortality and reproduction. Nature Ecology & Evolution.

[B14257388] Hijmans R (2024). _terra: Spatial Data Analysis_. https://CRAN.R-project.org/package=terra.

[B13952986] Ho L., Ané C. (2014). A linear-time algorithm for Gaussian and non-Gaussian trait evolution models. Systematic Biology.

[B13952995] Imarhiagbe E. E., Onyekwelu J. C., Olajide O. E. (2019). A review of the biodiversity conservation status in Nigeria. Journal of Wildlife and Biodiversity.

[B13953004] IUCN (2024). Background & History. International Union for Conservation of Nature’s Red List database1(3), Retrieved 11/04/2024 from.

[B13953013] IUCN (2025). Threats Classification Scheme (Version 3.3). International Union for Conservation of Nature’s Red List database 1(9), Retrieved 01/13/2025 from.

[B13953022] Ives A., Garland T. (2010). Phylogenetic logistic regression for binary dependent variables. Systematic Biology.

[B13953224] Kumschick S., Richardson D. M. (2013). Species‐based risk assessments for biological invasions: advances and challenges. Diversity and Distributions.

[B13953233] Lander J. (2014). R for everyone: Advanced analytics and graphics.

[B13953276] Luiselli L., Dendi D., Eniang E., Fakae B., Akani G., Fa J. (2019). State of knowledge of research in the Guinean forests of West Africa region. Acta Oecologica.

[B13953312] Maxwell S., Fuller R., Brooks T., Watson J. (2016). Biodiversity: The ravages of guns, nets and bulldozers. Nature.

[B13953321] Montero-Botey M., Kivuyo E., Sitati N., Perea R. (2024). Deforestation and water availability as main drivers of human-elephant conflict. Global Ecology and Conservation.

[B13953432] Paradis E., Claude J., Strimmer K. (2004). APE: Analyses of phylogenetics and evolution in R language. Bioinformatics.

[B13953352] Pebesma E. (2018). Simple Features for R: Standardized support for spatial vector data. The R Journal.

[B13953361] Penone C., Davidson A. D., Shoemaker K. T., Marco M., Rondinini C., Brooks T. M., Costa G. C. (2014). Imputation of missing data in life‐history trait datasets: Which approach performs the best?. Methods in Ecology and Evolution.

[B13953374] Pereira H., Navarro L., Martins I. (2012). Global biodiversity change: The bad, the good, and the unknown. Annual Review of Environment and Resources.

[B13953392] Rabosky D. L. (2016). Reproductive isolation and the causes of speciation rate variation in nature. Biological Journal of the Linnean Society.

[B13953401] Rhind S. (2009). Anthropogenic pollutants: a threat to ecosystem sustainability?. Philosophical Transactions of the Royal Society B: Biological Sciences.

[B13953410] Robbins M., Sawyer S. (2007). Intergroup encounters in mountain gorillas of Bwindi impenetrable National Park, Uganda. Behaviour.

[B13953441] Sesink C., Abwe E., Ambahe R., Anthony N., Fotso R., Locatelli S., Maisels F., Mitchell M., Morgan B., Pokempner A., Gonder M. (2015). Chimpanzee population structure in Cameroon and Nigeria is associated with habitat variation that may be lost under climate change. BMC Evol Biol.

[B13953457] Slatyer R. A., Hirst M., Sexton J. P. (2013). Niche breadth predicts geographical range size: a general ecological pattern. Ecology Letters.

[B13953476] Sol D., Timmermans S., Lefebvre L. (2002). Behavioural flexibility and invasion success in birds. Animal behaviour.

[B13953466] Soria C., Pacifici M., Marco M., Stephen S., Rondinini C. (2021). COMBINE: a coalesced mammal database of intrinsic and extrinsic traits. Ecology.

[B13953485] Ukpoju E., Abatan A., Obaedo B., Balogun O., Adegbite A. (2023). Assessing the effectiveness of biodiversity conservation strategies in Nigeria: A Comprehensive Review. International Journal of Applied Research in Social Sciences.

[B13953495] Upham N., Esselstyn J., Jetz W. (2019). Inferring the mammal tree: Species-level sets of phylogenies for questions in ecology, evolution, and conservation. PLoS Biol.

[B13953511] Warton D. I., Hui F. K. (2011). The arcsine is asinine: the analysis of proportions in ecology. Ecology.

[B13953520] Wickham H. (2009). ggplot2: Elegant graphics for data analysis.

[B13953528] Wickham H. (2014). Tidy Data. Journal of Statistical Software.

[B13953537] Wickham H., Francois R., Henry L., Muller K. (2023). dplyr: A grammar of data manipulation (R package version 1.1.2).. Retrieved from.

